# Anti-Inflammatory and Antiosteoclastogenic Activities of Parthenolide on Human Periodontal Ligament Cells *In Vitro*

**DOI:** 10.1155/2014/546097

**Published:** 2014-12-25

**Authors:** Xufang Zhang, Chen Fan, Yin Xiao, Xueli Mao

**Affiliations:** ^1^Department of Operative Dentistry and Endodontics, Guanghua School of Stomatology, Guangdong Province Key Laboratory of Stomatology, Sun Yat-sen University, Guangzhou 510055, China; ^2^Institute of Health and Biomedical Innovation, Queensland University of Technology, Brisbane, QLD 4059, Australia

## Abstract

Periodontitis is an inflammatory disease that causes osteolysis and tooth loss. It is known that the nuclear factor kappa B (NF-*κ*B) signalling pathway plays a key role in the progression of inflammation and osteoclastogenesis in periodontitis. Parthenolide (PTL), a sesquiterpene lactone extracted from the shoots of *Tanacetum parthenium*, has been shown to possess anti-inflammatory properties in various diseases. In the study reported herein, we investigated the effects of PTL on the inflammatory and osteoclastogenic response of human periodontal ligament-derived cells (hPDLCs) and revealed the signalling pathways in this process. Our results showed that PTL decreased NF-*κ*B activation, I-*κ*B degradation, and ERK activation in hPDLCs. PTL significantly reduced the expression of inflammatory (IL-1*β*, IL-6, and TNF-*α*) and osteoclastogenic (RANKL, OPG, and M-CSF) genes in LPS-stimulated hPDLCs. In addition, PTL attenuated hPDLC-induced osteoclastogenic differentiation of macrophages (RAW264.7 cells), as well as reducing gene expression of osteoclast-related markers in RAW264.7 cells in an hPDLC-macrophage coculture model. Taken together, these results demonstrate the anti-inflammatory and antiosteoclastogenic activities of PTL in hPDLCs *in vitro*. These data offer fundamental evidence supporting the potential use of PTL in periodontitis treatment.

## 1. Introduction 

Periodontitis is a highly prevalent condition in clinic and may lead to the destruction of alveolar bone and loss of teeth [1]. Lipopolysaccharide (LPS), a key pathogenic component of gram-negative bacteria, has been broadly reported to participate in periodontitis progression by inducing host cells to produce a wide range of proinflammatory cytokines [1]. Human periodontal ligament cells (hPDLCs) are a vital cell type involved in periodontitis as these cells maintain the homeostasis of periodontal tissues and regulate bone formation and resorption [2, 3]. In response to LPS, resident hPDLCs produce a range of inflammatory cytokines and matrix metalloproteinases (MMPs), including tumour necrosis factor-*α* (TNF-*α*), interleukin-1 (IL-1), IL-6, MMP-2, and MMP-9, which are responsible for activation of circulating immune cells and degradation of extracellular matrix [1, 4]. In addition, hPDLCs secrete osteoclastogenic-related cytokines, such as osteoprotegerin (OPG) and receptor activator of nuclear factor kappa B ligand (RANKL), which are essential in regulating alveolar bone destruction [5]. Furthermore, it has been reported that the inflammation-regulating properties of hPDLCs are triggered through the nuclear factor kappa B (NF-*κ*B) signalling pathway [6], indicating that NF-*κ*B may be a potential target for improving periodontitis therapies.

Parthenolide (PTL), a sesquiterpene lactone extracted from the shoots of* Tanacetum parthenium*, has been widely used to treat headache, migraine, and arthritis due to its anti-inflammatory properties [7]. In addition, PTL has also been reported to ameliorate endotoxic shock and prevent inflammation in immune glomerulonephritis [8, 9]. The anti-inflammatory properties of PTL may result from its NF-*κ*B inhibiting ability, which has been previously demonstrated in HeLa and macrophage cell lines [10, 11]. However, the effects of PTL on periodontitis-related cells have yet to be reported.

Based on previous studies of PTL, we hypothesized that PTL may inhibit LPS-induced signalling pathways and reduce inflammatory- and osteoclastogenic-related gene expression in hPDLCs. In the study reported herein, we investigated the effects of PTL on NF-*κ*B and extracellular signal-regulated kinases (ERK) signalling pathways in LPS-stimulated hPDLCs. In addition, the effects of PTL on LPS-induced inflammatory and osteoclastogenic-related gene expression in hPDLCs were investigated. Furthermore, the effects of PTL on hPDLCs-induced osteoclastogenesis in macrophages were determined using a Transwell coculture technique.

## 2. Methods

### 2.1. Cell Culture

hPDLCs and the murine-derived macrophage cell line RAW 264.7 cells were used in this study. Human teeth samples were obtained from consenting donors with ethical approval from the Queensland University of Technology. hPDLCs were isolated from healthy wisdom teeth from three different donors. The periodontal ligament was gently removed from the middle third of the root surface under sterile conditions. The tissue explants were then transferred to a primary culture flask and cultured in low glucose Dulbecco's Modified Eagle Medium (DMEM; Life Technologies, Australia) containing 10% (v/v) fetal bovine serum (FBS;* in vitro*, Australia) and 1% (v/v) penicillin/streptomycin (P/S; Life Technologies, Australia). The cells were allowed to grow out of the explant tissue in humidified air with 5% CO_2_ at 37°C, with fresh medium provided every 3 days. hPDLCs at passage 3 were used in the subsequent experiments.

RAW 264.7 cell cultures were maintained in DMEM supplemented with 10% FBS and 1% (v/v) P/S at 37°C in a humidified CO_2_ incubator. The cells were expanded through two passages before being used in the following experiments.

### 2.2. Cell Proliferation

The effects of PTL (Sigma-Aldrich, USA) on hPDLC proliferation were investigated using a 3-(4,5-dimethylthiazol-2-yl)-2,5-diphenyltetrazolium bromide (MTT) assay. hPDLCs were seeded at a density of 3 × 10^3^ cells/well in 96-well microplates and were allowed to adhere and spread for 12 h. The cells were then treated with various concentrations of PTL (1, 5, 10, and 20 *μ*M) for 1, 3, and 7 days, after which the cells were incubated with MTT solution (0.5 mg/mL, Sigma-Aldrich) at 37°C for 4 h. Dimethyl sulfoxide (DMSO; Sigma-Aldrich) was then used to dissolve the formazan crystals. The absorbance was measured at 495 nm using SpectraMax Microplate Reader (Molecular Devices, USA).

### 2.3. NF-*κ*B and ERK Signalling Pathways in hPDLCs in Response to PTL

The effects of PTL on the expression of NF-*κ*B, I-*κ*B, and ERK in hPDLCs were evaluated by Western blotting. hPDLCs were pretreated with 1 *μ*M PTL for 1 h and then stimulated with* Escherichia coli* LPS (1 *μ*g/mL, Sigma-Aldrich) for 15, 30, and 60 min. Whole cell lysates were collected in a lysis buffer containing 1% Ipegal, 1% sodium dodecyl sulphate, 50 mM Tris-HCl, 150 mM NaCl, and protease inhibitor cocktail (Roche, Swiss) at indicated time points. The protein concentration was determined by the bicinchoninic acid (BCA; Thermo Fisher Scientific, Australia) assay. Equal amounts of protein (10 *μ*g) were prepared and separated using 10% sodium dodecyl sulphate polyacrylamide gel electrophoresis (SDS-PAGE) and were then transferred onto nitrocellulose membranes (Pall Corporation, USA). The membranes were incubated with primary antibodies overnight at 4°C in Odyssey blocking buffer (LI-COR Biosciences, USA). Primary antibodies include anti-I-*κ*B, -NF-*κ*B, -ERK, and -p-ERK (1 : 1000) from Cell Signalling Technology, USA, and anti-GAPDH (1 : 1000) from Abcam, USA. The membranes were then washed twice in Tris-buffered saline containing 0.05% Tween-20 and then incubated with the corresponding fluorescent secondary antibodies (Cell Signalling Technology, USA) for 1 h at room temperature. Images were then captured and analysed using the Odyssey Infrared Imaging system and software (LI-COR Biosciences).

### 2.4. Matrix Metalloproteinase- (MMP-) 2 and MMP-9 Expression in PDLCs

The effects of PTL on the expression of MMP-2 and MMP-9 in hPDLCs were investigated at the gene and protein level. hPDLCs were pretreated with PTL (1 and 5 *μ*M) for 1 h and then stimulated with* Escherichia coli* LPS (1 *μ*g/mL, Sigma-Aldrich) for 1 and 3 days. Cells cultured in medium only served as the negative control, while cells cultured with LPS only served as the positive control. Cells were collected and subjected to Western blotting analysis using the same methods as described in Section 2.3. Primary antibodies include anti-MMP-2 and MMP-9 (1 : 1000) from Abcam, USA.

PTL-induced changes in hPDLC gene expression were determined using quantitative reverse transcriptase polymerase chain reaction (qRT-PCR). Primers for the target genes are listed in Table 1. Total RNA was collected and extracted using TRIzol reagent (Life Technologies). First strand cDNA was synthesized using the DyNAmo cDNA Synthesis Kit (Thermo Fisher Scientific, USA), as per the manufacturer's protocol. qRT-PCR was performed using SYBR reagent in an ABI 7500 Thermal Cycler (Applied Biosystems, Australia).

### 2.5. Anti-Inflammatory and Antiosteoclastogenic Activity of PTL

#### 2.5.1. Inflammatory- and Osteoclastogenic-Related Gene Expression in hPDLCs

The effects of PTL on the gene expression of inflammatory cytokines (IL-1, IL-6, and TNF-*α*) and osteoclastogenic-related factors (RANKL, OPG, and M-CSF) in hPDLCs were investigated by qRT-PCR as described in Section 2.4. Cells were also treated as described in the above section. Primers for the target genes are listed in Table 1.

#### 2.5.2. Coculture of hPDLCs and Macrophages

hPDLCs have been previously reported to regulate osteoclastic differentiation [2]. hPDLC-macrophage Transwell coculture technique was therefore employed to further investigate the effects of PTL on hPDLC-regulated macrophage-osteoclast differentiation [12]. Briefly, hPDLCs were seeded into the insert of the Transwell (Corning, USA) and treated with LPS containing different concentrations of PTL (0, 1, and 5 *μ*M) for 48 h. Wells containing medium only were included as the control. The medium was then discarded and murine macrophage RAW264.7 cells were then seeded into the lower chamber of the Transwell with fresh medium containing recombinant human soluble RANK Ligand (RANKL; 10 ng/mL, Chemicon, Australia). The macrophage RAW264.7 cells were then cocultured with hPDLCs for 7 days. Expression of osteoclastic genes (RANK, Calcitonin Receptor, Carbonic Anhydrase II, MMP-9, Cathepsin K, and TRAP) in RAW264.7 cells was then determined using qRT-PCR as described in Section 2.4 and hPDLC-induced osteoclasts were identified using tartrate-resistant acid phosphatase (TRAP) staining as described below.

#### 2.5.3. TRAP Staining

Following coculture with hPDLCs, RAW264.7 cells were fixed in a mixture of citrate (6.75 mM), acetone (65%), and formalin (3.7%). TRAP staining was performed using a TRAP staining assay kit (Sigma-Aldrich), as per manufacturer's instructions. TRAP-positive multinucleated cells (MNCs) containing three or more nuclei were regarded as osteoclasts. Images were captured using a Nikon ECLIPSE TS100 microscope (Nikon, Australia).

### 2.6. Statistical Analysis

All assays were performed in triplicate, with each treatment tested individually three times in cells from three different patients. All data were converted to the fold change of the control and one-way ANOVA with Student-Newman-Keuls (S-N-K) test was used to analyse the statistical difference. A *P* value of <0.05 was regarded as statistically significant.

## 3. Results

### 3.1. Effect of PTL on hPDLC Proliferation

**Figure 1:**
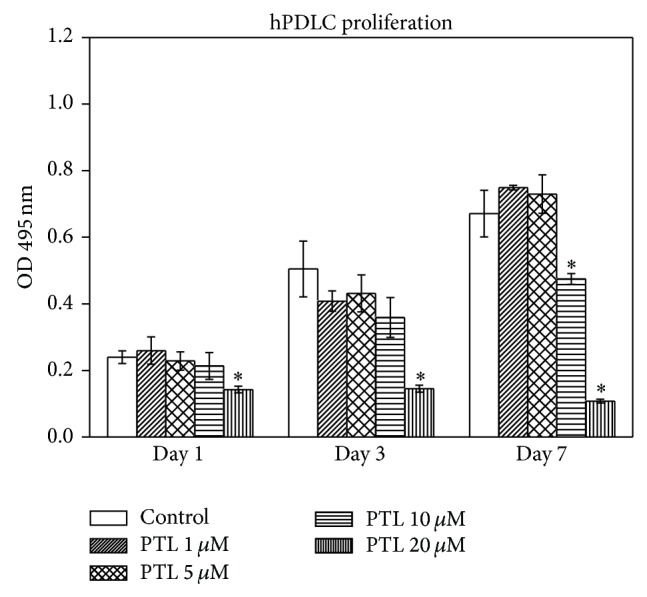
Effect of PTL on hPDLC proliferation. hPDLCs were exposed to PTL (1, 5, 10, and 20 *μ*M) for 1, 3, and 7 days. Cell proliferation was measured using the MTT assay. The data are expressed as the percentage of the control (containing medium only). Error bars indicate mean ± SEM (*n* = 3). ^*^ *P* < 0.05 versus the control.

To identify the effects of PTL on cell proliferation, hPDLCs were treated with different concentrations of PTL (1, 5, 10, and 20 *μ*M) for 1, 3, and 7 days (Figure 1). As shown in Figure 1, no effects of PTL at 1 and 5 *μ*M on hPDLC proliferation were detected compared to the control (*P* > 0.05). However, 10 *μ*M PTL reduced hPDLC proliferation by 29.3% ± 2.4% at day 7 compared to the control (*P* < 0.05). In addition, hPDLC proliferation was 40.6% ± 4.2%, 71.2% ± 2.1%, and 83.9% ± 0.8% below the control (*P* < 0.05), when exposed to 20 *μ*M PTL for 1, 3, and 7 days, respectively. Taken together, these data indicate that PTL inhibits hPDLC proliferation in a dose-dependent manner, notwithstanding the fact that PTL at 1 and 5 *μ*M has no inhibitory effects on hPDLC proliferation.

### 3.2. Effects of PTL on Signalling Pathways in hPDLCs

**Figure 2:**
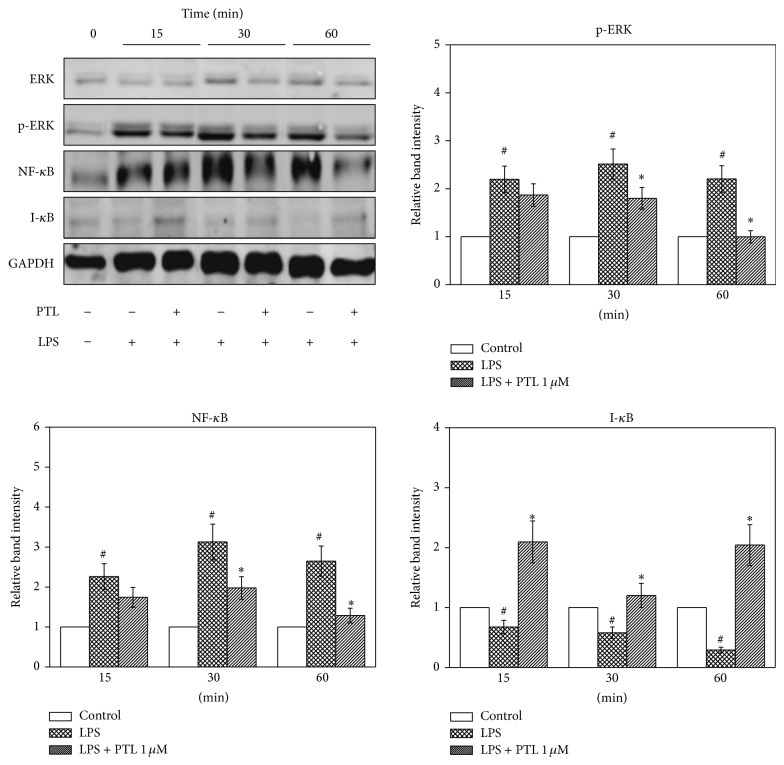
Effects of PTL on signalling pathways in hPDLCs. hPDLCs were pretreated with PTL 1 *μ*M for 1 hour and then stimulated by LPS for 15, 30, and 60 min. Protein abundance was determined by Western blot. Error bars indicate mean ± SEM (*n* = 3). ^#^ *P* < 0.05 versus the blank group while ^*^ *P* < 0.05 versus the LPS group.

The effects of PTL on the expression of NF-*κ*B, I-*κ*B, and ERK in hPDLCs were evaluated as they play vital roles in mediating the inflammation process in periodontitis [6]. As shown in Figure 2, expression of NF-*κ*B/p50 and p-ERK were significantly increased, while I-*κ*B was decreased in hPDLCs when exposed to LPS for 15, 30, and 60 min compared to the control (*P* < 0.05). However, PTL downregulated p-ERK and NF-*κ*B/p50 expression at 30 and 60 min and upregulated I-*κ*B expression, at 15, 30, and 60 min in hPDLCs compared to LPS-treated cells (*P* < 0.05). In summary, these data illustrated that PTL reduced p-ERK and NF-*κ*B/p50 abundance, and it also increased I-*κ*B expression in hPDLCs.

### 3.3. Effects of PTL on the Protein and Gene Expression of MMP-2 and MMP-9

**Figure 3:**
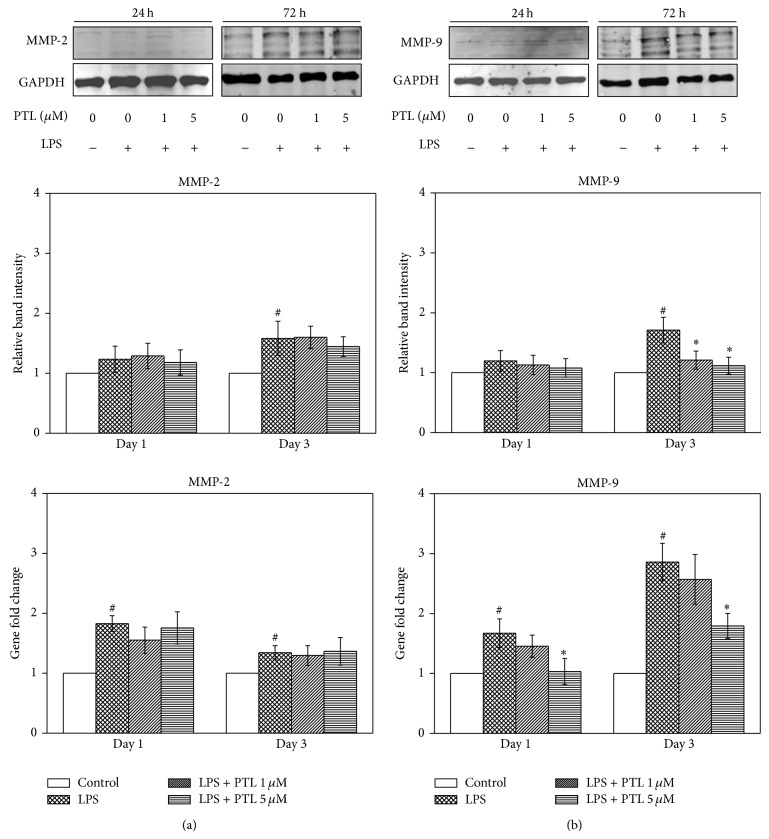
Effect of PTL on the expression of MMP-2 and MMP-9. (a) Expression of MMP-2; (b) expression of MMP-9. hPDLCs were treated with PTL (1 and 5 *μ*M) for 1 and 3 days and then total protein and RNA were collected. The abundance of proteins was detected by Western blot. GAPDH was included as a loading control. The intensities of the bands were measured with densitometry and first normalized to GAPDH and then further converted to the percentage of the control (containing medium only). Error bars indicate mean ± SEM (*n* = 3). ^#^ *P* < 0.05 versus the control while ^*^ *P* < 0.05 versus the LPS group.

MMP-2 and MMP-9 have been demonstrated to regulate proteolytic degradation in periodontitis [4]; the effects of PTL on the expression of MMP-2 (Figure 3(a)) and MMP-9 (Figure 3(b)) in hPDLCs were therefore investigated. As is illustrated in Figure 3, LPS increased MMP-2 and MMP-9 protein abundance at day 3 as well as gene expression at both day 1 and day 3 in hPDLCs compared to the control (*P* < 0.05). However, no effect of PTL on MMP-2 protein abundance or gene expression was observed. Indeed, MMP-9 protein expression was below the LPS group (*P* < 0.05) when exposed to 1 or 5 *μ*M PTL for 3 days. In addition, 5 *μ*M PTL significantly reduced MMP-9 gene expression at day 1 and day 3 compared to the LPS group (*P* < 0.05). These results indicate that PTL downregulates MMP-9 protein abundance and gene expression in hPDLCs but has no effect on the expression of MMP-2.

### 3.4. Effects of PTL on the Expression of Inflammatory and Osteoclastogenic Genes

**Figure 4:**
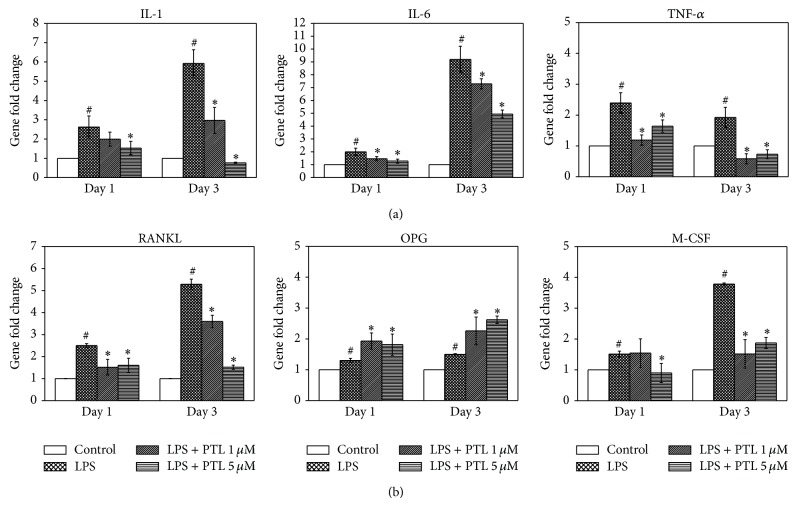
Effect of PTL on hPDLC gene expression. (a) Expression of inflammatory genes (IL-1*β*, IL-6, and TNF-*α*); (b) expression of osteoclastogenic genes (M-CSF, RANKL, and OPG). hPDLCs were treated with PTL (1 and 5 *μ*M) for 1 and 3 days and then total RNA was collected. After RNA extraction, first strand cDNA was synthesized. The cDNA sample was then amplified using qRT-PCR. The expression of the target gene was first normalized to 18S and then further converted to the percentage of the control (containing medium only). Error bars indicate mean ± SEM (*n* = 3). ^#^ *P* < 0.05 versus the control while ^*^ *P* < 0.05 versus the LPS group.

Based on the MTT assay results, expression of inflammatory genes (IL-1, IL-6, and TNF-*α*; Figure 4(a)) and osteoclastogenic genes (RANKL, OPG, and M-CSF; Figure 4(b)) in hPDLCs was determined after exposure to PTL at 1 and 5 *μ*M for 1 and 3 days. The expression of all genes was upregulated compared with the control (*P* < 0.05) when exposed to LPS for either 1 or 3 days. However, 5 *µ*M PTL significantly reduced the expression of IL-1, IL-6, TNF-*α*, RANKL and M-CSF genes, and it also increased OPG gene expression (compared to LPS group, *P* < 0.05). With the exception, 1 *μ*M PTL had no effect on IL-1 and M-CSF expression at day 1; 1 *μ*M PTL also induced the above changes in gene expression in hPDLCs. In summary, these results indicate that PTL inhibits inflammation- and osteoclastogenesis-related gene expression in hPDLCs after stimulation with LPS.

### 3.5. PTL Attenuates hPDLC-Induced Osteoclastogenic Differentiation in Macrophages

**Figure 5:**
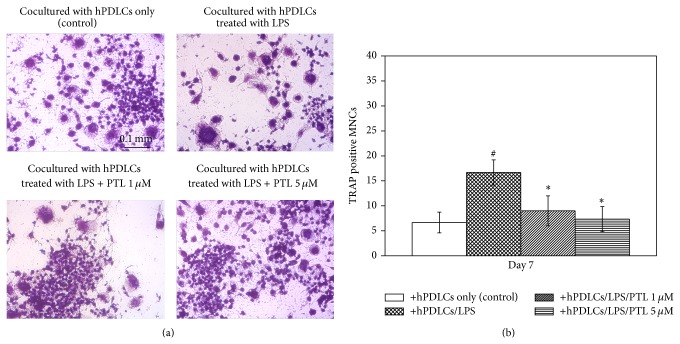
TRAP staining of osteoclasts cocultured with hPDLCs. (a) TRAP-positive staining in RAW 264.7 cells. TRAP staining of RAW 264.7 cells was performed using assay kit. Images were captured using a Nikon ECLIPSE TS100 microscope with a 10x camera. Representative images are depicted from three patients. (b) Percentage of TRAP-positive MNCs in RAW 264.7 cells. Three randomly selected images were recorded in each treatment group and the number of MNCs was counted. The final data was the average cell number of nine different images from three different patients. Error bars indicate mean ± SEM (*n* = 3). ^#^ *P* < 0.05 versus the control while ^*^ *P* < 0.05 versus the LPS group.

hPDLCs have been reported to influence osteoclastogenic differentiation in RAW264.7 cells [12]. TRAP staining was therefore used to identify the effects of PTL on hPDLC-induced osteoclastogenic differentiation in RAW264.7 cells (Figure 5) as TRAP is highly expressed in osteoclasts. A higher percentage of MNCs (osteoclasts) were detected in RAW264.7 cells when cocultured with LPS-treated hPDLCs compared to the control group. However, decreased MNCs were observed in RAW264.7 cells when cocultured with PTL- and LPS-treated hPDLCs compared to the hPDLCs treated with LPS alone. These results demonstrate that PTL inhibits hPDLC-induced osteoclastogenic differentiation in RAW264.7 cells.

### 3.6. Effects of PTL on the Expression of Genes in Osteoclasts

**Figure 6:**
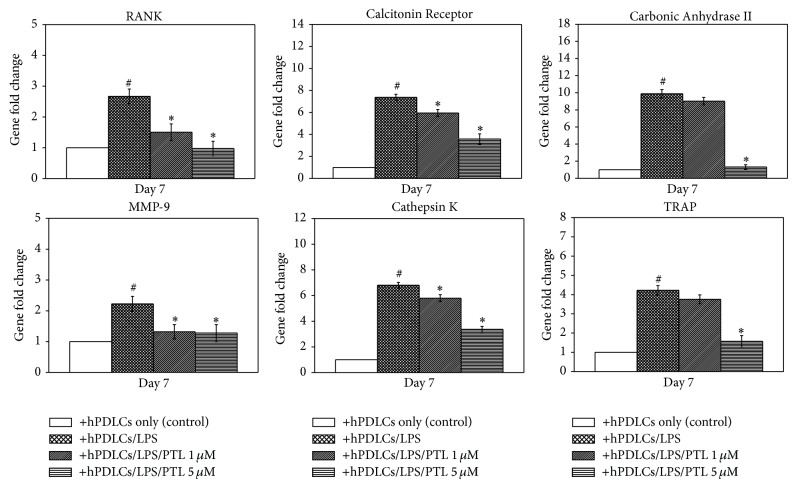
Effect of PTL on osteoclast gene expression. After coculturing with differently treated hPDLCs, total RNA was collected from RAW 264.7 cells. Gene expression was measured by RT-PCR and the expression of the target gene was first normalized to *β*-actin and then further converted to the percentage of the control (coculture with hPDLCs only). Error bars indicate mean ± SEM (*n* = 3). ^#^ *P* < 0.05 versus the control while ^*^ *P* < 0.05 versus the LPS-treated hPDLCs group.

To further identify the effects of PTL on osteoclastogenic differentiation in RAW264.7 cells, expression of osteoclast-related genes, including RANK, Calcitonin Receptor, Carbonic Anhydrase II, MMP-9, Cathepsin K, and TRAP, was investigated following coculture with treated hPDLCs (Figure 6). Expression of all genes listed was increased in RAW264.7 cells when they were cocultured with LPS-treated hPDLCs, compared to the control (*P* < 0.05); however, PTL- (5 *μ*M) and LPS-treated hPDLCs significantly decreased the expression of those genes compared to the hPDLCs treated with LPS alone (*P* < 0.05). In addition, PTL- (1 *μ*M) and LPS-treated hPDLCs downregulated RANK, Calcitonin Receptor, MMP-9, and Cathepsin K gene expression compared to the cells treated with LPS alone (*P* < 0.05). These data provide further evidence supporting the osteoclastogenesis-inhibiting ability of PTL.

## 4. Discussion

Bacteria-induced inflammation and osteolysis are the most important pathological conditions in periodontitis [1]. Critically, current strategies to prevent or treat periodontitis are far from satisfactory. Numerous reports have focused on the key role of hPDLCs in periodontitis, as they produce inflammatory mediators and induce osteoclast differentiation in response to bacterial pathogens [2, 5]. PTL, an active constituent of the plant* Tanacetum parthenium*, has been shown to have potential in the treatment of inflammatory diseases [8, 9]. We report above, for the first time, that PTL inhibits the production of inflammatory cytokines, MMPs, and osteoclastogenic cytokines in LPS-stimulated hPDLCs, as well as suppressing hPDLC-induced osteoclastogenic differentiation of macrophages.

The transcriptional factor NF-*κ*B has been found to play essential roles in regulating the expression of LPS-induced inflammatory cytokines, such as IL-1, IL-6, IL-8, TNF-*α*, and RANKL [10, 13]. These cytokines, in turn, contribute to osteoclast activation and bone destruction in periodontitis. Previous studies have shown that blockade of TNF-*α* and RANKL by neutralizing antibodies only partially suppresses LPS-induced osteoclastogenesis [14]. Therefore, a more reasonable approach is to inhibit the NF-*κ*B pathway which is responsible for their production. In most cell types, NF-*κ*B is bound to its inhibitor I-*κ*B and resides in the cytoplasm as an inactive NF-*κ*B/I-*κ*B complex [13]. The activated form of NF-*κ*B is a heterodimer of the p65 subunit associated with either the p50 or the p52 subunit, and then the activated NF-*κ*B migrates into the nucleus and initiates the expression of inflammatory genes [13]. Our results demonstrate that PTL inhibits the NF-*κ*B signalling pathway in LPS-stimulated hPDLCs via decreasing NF-*κ*B p50 activation and I-*κ*B degradation. The NF-*κ*B-inhibiting ability of PTL may therefore support the role for its anti-inflammatory potential in periodontitis. Other studies also found that PTL directly binds to I-*κ*B kinase in the HeLa cell line and inhibits p65 translocation in macrophages and osteoclasts [8–11]. However, the exact target of PTL has to be confirmed in future studies.

Besides the NF-*κ*B signalling pathway, mitogen-activated protein kinases (MAPKs), including ERKs, c-Jun N-terminal kinases (JNKs), and p38 MAPK also play important roles in mediating inflammation-related signalling pathways [15]. It has been reported that IL-1 induced RANKL expression in hPDLCs is mediated by the phosphorylation of ERK [16]. In addition, the ERK inhibitor but not JNK or p38 MAPK inhibitor has been shown to attenuate force-mediated stimulation of NF-*κ*B-DNA binding in hPDLCs [15], indicating the importance of ERK signalling in periodontitis and its correlation with NF-*κ*B. Furthermore, a previous study has reported that PTL reduced IL-8 production by inhibiting both ERK and NF-*κ*B pathways [17]. Our study suggests that PTL inhibits LPS-induced phosphorylation of ERK in hPDLCs, demonstrating the possible mechanisms of the anti-inflammatory effects of PTL on hPDLCs.

MMP-2 and MMP-9, also called type IV collagenases or gelatinases, are involved in the digestion of bone collagen [4]. Indeed, MMP-9 levels in gingival crevicular fluid have been used to identify the stage of periodontitis [4]. PTL and its derivations have been shown to inhibit MMP-9 expression in breast cancer cells and prevent tumour metastasis [18]. Our study also demonstrated that PTL suppressed MMP-9 gene expression and protein abundance, indicating the potential of PTL in preventing matrix degradation in periodontitis. One possible underlying mechanism might be via PTL inhibiting the NF-*κ*B signalling pathway. It is worth noting that PTL had no significant influence on LPS-induced MMP-2 production. It is known, however, that the transcriptional promoters of MMP-2 and MMP-9 are distinct, albeit they are in fact functionally related to each other [19]. There is a range of transcriptional factors, including NF-*κ*B, AP-1, PEA3, Sp-1, and *β*-catenin/Tcf-4, which can regulate the expression of MMP genes due to several* cis*-elements in the promoters of MMPs [19]. Our results nevertheless indicate that inhibition of NF-*κ*B signalling might be not sufficient to abrogate MMP-2 production.

The importance of inflammatory cytokines in periodontitis has been well documented. It has been reported that IL-1 and TNF-*α* antagonists inhibit the inflammatory responses and bone destruction in experimental periodontitis [20, 21]. PTL has also been previously reported to inhibit inflammatory cytokines (IL-1, IL-6, and TNF-*α*), chemokine (IL-8), and cyclooxygenase in macrophages and epithelium cells [22, 23]. PTL also attenuates LPS-induced systemic inflammation by reducing circulating IL-6 and TNF-*α* in animal model [24, 25]. Consistent with previous studies, our results demonstrate that LPS upregulates IL-1, IL-6, and TNF-*α* gene expression in hPDLCs, whereas pretreatment with PTL effectively inhibits the production of these cytokines, thus indicating the anti-inflammatory potential of PTL in treatment of periodontitis. Furthermore, the inhibitory effect of PTL on LPS-induced IL-1, IL-6, and TNF-*α* synthesis could, at least in part, contribute to the downregulation of matrix degradation and osteoclastogenesis, for all these cytokines participate in the production of metalloproteinases and osteoclastogenic factors of RANKL and M-CSF [26].

In periodontal tissue RANKL, M-CSF and OPG are essential regulators of alveolar bone resorption [1, 27]. RANKL controls osteoclast differentiation and activation via binding to its receptor (RANK) which is located on the cell surface of osteoclast precursors [28]. OPG is a RANKL “decoy” receptor preventing osteoclast differentiation [28]. A decreased ratio of OPG/RANKL has been reported in periodontal tissue with periodontitis [1]. Further, the OPG/RANKL system has been reported to play essential roles in regulating hPDLC-induced osteoclast differentiation [1]. M-CSF is another essential factor for osteoclast formation through binding to the M-CSF receptor on osteoclast surface and activation of the Akt signalling pathway [29]. In this study, we found that PTL increased antiosteoclastogenic gene of OPG and attenuated expression of the osteoclastogenic genes RANKL and M-CSF in LPS-stimulated hPDLCs, indicating antiosteoclastogenesis potential of PTL in periodontal diseases. In addition, as NF-*κ*B signaling is the key regulator of inflammation and osteoclastogenesis, the inhibitory effect of PTL on NF-*κ*B pathway might contribute to the downregulation of inflammatory- and osteoclastogenic-related genes in hPDLCs.

As previous studies have demonstrated, PTL could directly inhibit osteoclastogenic differentiation. Yip et al. applied PTL directly to cell culture of macrophages and found that PTL inhibits osteoclastogenesis of macrophage and osteoclastic bone resorption [10]. In contrast, our study focuses on the effect of PTL on hPDLCs. It is well known that hPDLCs could secret soluble cytokines that influence osteoclast differentiation and activity. Our previous data showed that PTL inhibits cytokines production of hPDLCs. Subsequently, we aimed to clarify whether PTL-treated hPDLCs could suppress osteoclasts differentiation of macrophages. Therefore, a Transwell coculture technique using RAW264.7 cells and hPDLCs was employed in this study. In our coculture study, osteoclast differentiation related markers, including RANK [28] and Calcitonin Receptor [30], were reduced in RAW264.7 cells when they were cocultured with PTL-treated hPDLCs. Moreover, genes regulating bone resorption of osteoclasts (Cathepsin K, MMP-9, Carbonic Anhydrase II, and TRAP [30, 31]) were reduced when they were cocultured with PTL-treated hPDLCs. These results indicate that PTL could possibly inhibit osteoclastogenic differentiation of RAW264.7 cells by regulating the production of soluble factors (such as RANKL, OPG, and M-CSF) in hPDLCs. In other words, PTL could suppress osteoclastogenic differentiation indirectly by influencing hPDLCs. This finding offers further evidence supporting the potential of PTL as an antiresorption therapy in periodontitis.

Conventional treatment for periodontitis, including mechanical scaling or disinfection therapies, is generally successful in eliminating bacterial infection. However, it remains a challenge to completely halt the inflammatory process and prevent bone resorption. In this study, we found that PTL reduced the inflammatory- and osteoclastogenic-related gene expression in LPS-stimulated hPDLCs. These findings provide fundamental evidence supporting further investigation on the effect of PTL in periodontitis using animal models. Clinically, PTL possesses great potential to be used as complementary medicine to control inflammation and bone resorption in periodontitis. For instance, PTL could be incorporated into a hydrogel or periodontal dressing paste, which can be applied to periodontal lesions following scaling treatment or periodontal flap operation. Further studies are needed to optimize the formulation of PTL and to clarify its pharmacokinetic properties in preparation for* in vivo* studies and clinical use.

**Figure 7:**
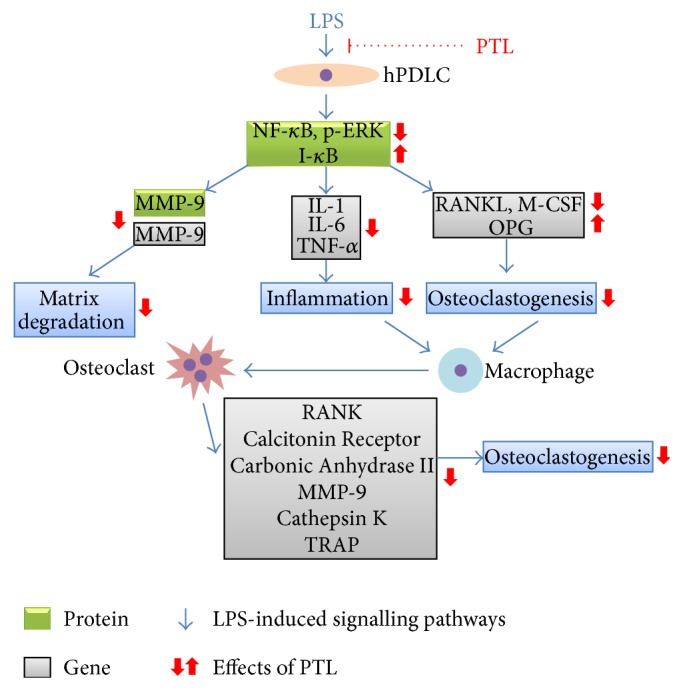
The underlying mechanisms of the effects of PTL treatment on hPDLCs and RAW 264.7 cells.

In summary, this study has shown that PTL inhibits the activation of NF-*κ*B and ERK signalling pathways, as well as the expression of inflammatory and osteoclastogenic genes in LPS-stimulated hPDLCs* in vitro*. Additionally, PTL suppresses osteoclast differentiation of macrophages induced by LPS-stimulated hPDLCs. The possible mechanisms for the inhibitory effects of PTL on the inflammation and osteoclastogenesis of hPDLCs were summarised in Figure 7. Our overall findings provide fundamental evidence supporting the premise that PTL has the potential to be utilized as a supplement medicine for the current therapeutic treatment of periodontitis.

## Figures and Tables

**Table 1:** Primers used in qRT-PCR.

Gene: *Homo sapiens* (H) & *Mus musculus* (M)	Primers: forward (F) & reverse (R)
IL-1 (H)	F: 5′-TTACAGTGGCAATGAGGATGAC-3′
	R: 5′-TGCTGTAGTGGTGGTCGGAGA-3′

IL-6 (H)	F: 5′-AGGAGACTTGCCTGGTGAAA-3′
	5′-CAGGGGTGGTTATTGCATCT-3′

TNF-*α* (H)	F: 5′-CCTGGTATGAGCCCATCTATC-3′
	R: 5′-GGTTGGATGTTCGTCCTCCTC-3′

RANKL (H)	F: 5′-AGAGCAGAGAAAGCGATGGTG-3′
	R: 5′-GAACCAGATGGGATGTCGGT-3′

OPG (H)	F: 5′-CGCTCGTGTTTCTGGACATCT-3′
	R: 5′-CACACGGTCTTCCACTTTGC-3′

M-CSF (H)	F: 5′-AGCATGACAAGGCCTGCGTC-3′
	R: 5′-AAGCTGTTGTTGCAGTTCTTGC-3′

MMP-2 (H)	F: 5′-CCGTCGCCCATCATCAA-3′
	R: 5′-AGATATTGCACTGCCAACTCT-3′

MMP-9 (H)	F: 5′-TCGTGGTTCCAACTCGGTTT-3′
	R: 5′-GCGGCCCTCGAAGATGA-3′

18s (H)	F: 5′-TTCGGAACTGAGGCCATGAT-3′
	R: 5′-CGAAC CTCCGACTTCGTTC-3′

RANK (M)	F: 5′-GCAGCTCAACAAGGATACGG-3′
	R: 5′-GGTGCAGTTGGTCCAAGGTT-3′

Calcitonin Receptor (M)	F: 5′-CTTAGCTGCCAGAGGGTGAC-3′
	R: 5′-TGCAACTTATAGGATCCCGCC-3′

Carbonic Anhydrase II (M)	F: 5′-AGCAGCGAGCAGATGTCTC-3′
	R: 5′-TGAGCTGGACGCCAGTTG-3′

MMP-9 (M)	F: 5′-GGGCGTGTCTGGAGATTCG-3′
	R: 5′-CACCTGGTTCACCTCATGGTC-3′

Cathepsin K (M)	F: 5′-CCAGTTTTACAGCAGAGGTGTG-3′
	R: 5′-CTTGCTTCCCTTCTGGGTG-3′

TRAP (M)	F: 5′-CACTCCCACCCTGAGATTTGT-3′
	R: 5′-CATCGTCTGCACGGTTCTG-3′

*β*-actin (M)	F: 5′-CATACCCAAGAAGGAAGGCTGG-3′
	R: 5′-GCTATGTTGCTCTAGACTTCGAGC-3′

## Abbreviations

MTT: 3-(4,5-Dimethylthiazol-2-yl)-2,5-diphenyltetrazolium bromide assay
BCA: Bicinchoninic acid
DMSO: Dimethyl sulfoxide
ERK: Extracellular signal-regulated kinases
hPDLCs: Human periodontal ligament-derived cells
IL: Interleukin
LPS: Lipopolysaccharide
MAPKs: Mitogen-activated protein kinases
MMP: Matrix Metalloproteinases
M-CSF: Macrophage colony stimulating factor
MNCs: Multinucleated cells
NF-*κ*B: Nuclear factor kappa B
OPG: Osteoprotegerin
PTL: Parthenolide
qRT-PCR: Quantitative reverse transcriptase polymerase chain reaction
RANK: Receptor activator of NF-kappa B
RANKL: Receptor activator of NF-kappa B ligand
SDS-PAGE: Sodium dodecyl sulphate polyacrylamide gel electrophoresis
TRAP: Tartrate-resistant acid phosphatase
TNF-*α*: Tumour necrosis factor-*α*.
